# MicroRNAs as Biomarkers for Nephrotic Syndrome

**DOI:** 10.3390/ijms22010088

**Published:** 2020-12-23

**Authors:** Kenji Tsuji, Shinji Kitamura, Jun Wada

**Affiliations:** Department of Nephrology, Rheumatology, Endocrinology and Metabolism, Okayama University Graduate School of Medicine, Dentistry and Pharmaceutical Sciences, 2-5-1 Shikata-cho, Okayama 700-8558, Japan; gmd422036@s.okayama-u.ac.jp (K.T.); junwada@okayama-u.ac.jp (J.W.)

**Keywords:** microRNA, nephrotic syndrome, biomarker, minimal change nephrotic syndrome, focal segmental glomerulosclerosis, membranous glomerulonephropathy

## Abstract

Nephrotic syndrome represents the clinical situation characterized by presence of massive proteinuria and low serum protein caused by a variety of diseases, including minimal change nephrotic syndrome (MCNS), focal segmental glomerulosclerosis (FSGS) and membranous glomerulonephropathy. Differentiating between diagnoses requires invasive renal biopsies in general. Even with the biopsy, we encounter difficulties to differentiate MCNS and FSGS in some cases. There is no other better option currently available for the diagnosis other than renal biopsy. MicroRNAs (miRNAs) are no-coding RNAs of approximately 20 nucleotides in length, which regulate target genes in the post-transcriptional processes and have essential roles in many diseases. MiRNAs in serum and urine have been shown as non-invasive biomarkers in multiple diseases, including renal diseases. In this article, we summarize the current knowledge of miRNAs as the promising biomarkers for nephrotic syndrome.

## 1. Introduction

Nephrotic syndrome (NS) is defined as massive proteinuria and low serum total protein due to the disrupted function of glomerular filtration barrier [1,2]. It may cause multiple metabolic effects and complications, including hypercoagulability, bacterial infection and acute kidney injury [2]. The underlying pathological etiologies of NS are diverse. For younger ages, the etiology of NS is more likely to be from minimal change nephrotic syndrome (MCNS) and focal segmental glomerulosclerosis (FSGS), while there are varieties of etiologies for older ages, including MCNS, FSGS, membranous glomerulonephropathy (MGN) and others [3,4]. Accumulating evidence suggests that MCNS and FSGS may be due to the circulating factors [5,6]. For example, Sharma et al. reported that a single injection of plasma obtained from FSGS patients into rats caused transient proteinuria, suggesting that circulating factors might be involved in the pathogenesis in FSGS [7]. Wei et al. reported that soluble form of the urokinase receptor (suPAR), which was shown to activate podocyte integrin β3, might be the cause of FSGS [8]. However, it is still controversial whether suPAR is the pathogenetic of FSGS, and exact pathophysiology of FSGS and MCNS are still uncertain. In the point of prognosis of NS, patients with MCNS are generally steroid sensitive, while patients with FSGS are more likely to be steroid resistant. Although renal biopsy is the standard method to differentiate these diseases especially in adult patients, it is an invasive examination with possible complications. In addition, it is difficult to make the accurate diagnosis in some cases, especially because the glomerular segmental sclerosis in FSGS patients is focal and sometimes patients need repeated biopsies for the accurate diagnosis. Therefore, non-invasive biomarkers may be used to differentiate between different etiologies of NS. In addition, ideal biomarkers also reflect disease activity for monitoring response to treatment, progression and determining disease prognosis.

MicroRNAs (miRNAs) are single-stranded non-coding RNAs, which are an average of 22 nucleotides in length, and which regulate target genes in the post-transcriptional processes [9,10]. In mammals, more than a thousand different miRNAs have been identified [9,10] and these miRNAs are reported to be involved in multiple biological cellular and molecular processes, including cell proliferation, apoptosis and differentiation [11]. In most cases, miRNAs interact with three prime untranslated regions (3′-UTR) in the target genes, which results in the degradation of mRNAs or translational inhibition [12]. Aberrant expression of miRNAs has been reported to be associated with human diseases [12,13]. miRNAs have been reported to be secreted into biological fluid, including serum and urine. miRNAs are also carried in the extracellular vesicles, including exosomes and microvesicles, which are transferred into recipient cells, in which gene translations are regulated as the cell–cell communication. miRNAs have been widely reported as promising biomarkers for many diseases. In fact, they have been shown as non-invasive biomarkers in multiple diseases including cancers and renal diseases [14,15,16,17]. Because miRNAs may directly be associated with pathogenesis of some types of NS, to analyze miRNA profiles in NS patients is not only for the diagnosis for the specific diseases, but also for understanding and elucidating the disease pathogenesis and even for establishing novel therapies. There is accumulating evidence for novel and non-invasive biomarkers of miRNAs as liquid biopsy in NS. In this review article, we summarize the current knowledge about miRNAs as promising biomarkers for NS.

## 2. miRNAs in Nephrotic Syndrome

There are several reports that have analyzed miRNAs in NS patients (Table 1). Luo et al. analyzed serum and urine miRNAs in child idiopathic NS patients and indicated increased levels in serum miR-30a-5p, miR-151-3p, miR-150, miR-191 and miR-19b as well as urine miR-30a-5p in NS patients compared to healthy controls [18]. Zhang et al. also analyzed child NS patients and reported the increased levels of peripheral blood miR-17-5p in child NS patients compared to healthy controls [19]. Chen et al. analyzed urine samples from child NS patients by high-throughput illumina sequencing via synthesis technology and reported that levels of urine miR-194-5p, miR-146b-5p, miR-378a-3p, miR-23b-3p and miR-30a-5p were higher compared to age and sex-matched healthy controls, and these miRNAs were decreased during the clinical remission period [20]. They also indicated that levels of urine miR-194-5p and miR-23b-3p were positively correlated with levels of urine protein, and proposed that urine miR-194-5p and miR-23b-3p can be the potential biomarkers for diagnosis and monitoring in NS patients. Wang el al. reported that levels of serum miR-503 in child NS patients measured by qPCR were lower than in healthy controls [21]. They used rat mesangial cells to explore the effect of miR-503, and indicated that miR-503 may contribute to the aberrant proliferation by targeting cyclin E, which was found to be the target of miR-503 by luciferase reporter assay. In addition to the analysis of biological fluid, kidney tissues have also been applied for the analysis of miRNAs. Lu et al. analyzed kidney tissues from child NS patients in different subtypes, and indicated that miR-191 levels were higher and miR-151-3p levels were lower in all NS subtypes compared to the controls [22]; that is the different trend as increased levels in both miR-191 and miR-151-3p under the analysis of serum [18]. These differences might be because of the different regulatory mechanism between expression in tissue and secretion into blood or urine. Further exploration might uncover the pathophysiology of podocyte injury and disease mechanism of NS.

In the adult study, Wang et al. analyzed the urine sediment from patients with NS caused by diabetes nephropathy (DN), MCNS, FSGS and MGN, and reported that the levels of urine miR-638 in NS patients were lower than non-nephrotic controls, and that urine miR-29a, miR-192 and miR-200c levels were significantly different between diagnostic groups [23]. Specifically, urine miR-200c levels in MCNS and FSGS patients were higher than patients with other diagnosis, while urine miR-192 levels were lower in DN patients compared to patients with other diagnosis. Zapata-Benavides et al. reported that serum miR-16-1 levels in NS patients analyzed by qPCR were lower than healthy controls [24]. Teng et al. also investigated the miRNA profile using serum samples with qPCR verification and reported that serum miR-181a, miR-210, miR-30a, miR-942, miR-192 and miR-586 levels were upregulated in NS patients [25]. In addition, serum miR-30a levels in treatment-resistant NS patients were higher than treatment-sensitive NS patients, indicating the use of miR-30a levels to differentiate between pathological subtypes or predict prognosis. Another report using qPCR indicated the increase in serum miR-181a levels in NS patients compared to healthy controls [26].

This increase and/or decrease in miRNAs might be the reflection of podocyte injury or associated with pathophysiology of the diseases. It is reported that podocyte cytoskeleton is regulated by several miRNAs, including miR-30, miR-132, miR-134 and miR-29a [27]. The increase in serum and urine miR-30a-5p levels in child NS might be associated with the disorder of podocyte cytoskeleton [18,20]. Although the changes in the expression of these miRNAs in NS patients have been shown, further reports are required to differentiate the causes of NS, such as MCNS, FSGS and MGN. Next, we summarize the miRNA analysis of specific type of NS.

## 3. miRNAs in Membranous Glomerulonephropathy

MGN is one of the important causes of NS, especially for adults. Although the pathogenesis of MGN is not fully understood, recent studies revealed the autoantibodies to the M-type receptor for phospholipase A2 receptor 1 (PLA2R1) [28], thrombospondin type-1 domain-containing 7A (THSD7A) and neural epidermal growth factor-like 1 protein (NELL-1) as the pathogenesis of MGN [28,29,30]. PLA2R1 was found to be expressed on podocytes in normal human glomeruli and to be colocalized with IgG4 in immune deposits in glomeruli from MGN patients, indicating that PLA2R1 may be a major antigen in MGN [28]. Similarly, THSD7A, also found to be expressed on normal podocytes, was shown to be highly expressed in podocyte that was colocalized with IgG4 in MGN patients without anti-PLA2R1 antibodies [30]. In addition, IgG eluted from the frozen kidney in a MGN patient with positive anti-THSD7A antibodies specifically recognized recombinant THSD7A by immunoblot analysis, indicating that THSD7A might be involved in the pathogenesis of MGN [30]. More recently, NELL-1 was shown to be involved in the pathogenesis of MGN negative for both PLA2R1 and THSD7A antibodies. Unlike, PLA2R1 and THSD7A, NELL-1 is barely detectable in normal glomeruli, but is highly expressed in NELL-1-associated MGN podocytes, colocalizing with IgG [29]. In addition, immunoblot analysis revealed the reactivity to NELL-1 in sera from NELL-1-associated MGN, indicating the presence of anti-NELL-1 antibodies. Therefore, monitoring circulating antibodies for these markers might be the useful option for the diagnosis or monitoring in patients with MGN. Indeed, Guo et al. reported that more than 70% idiopathic MGN patients were positive for PLA2R antibody and that the titer of serum anti-PLA2R antibody was correlated with urine proteinuria and serum albumin levels of pre- and post- therapeutic values in idiopathic MGN patients, suggesting that serum anti-PLA2R antibody may guide clinical diagnosis and treatment as well as the prognostic clues [31]. Nevertheless, quite a few patients with primary MGN are negative for PLA2R, THSD7A and NELL-1 antibodies; thus, further exploration for finding other biomarkers, including miRNAs, is still required.

There are a limited number of publications of miRNAs in MGN patients (Table 2). Chen et al. analyzed the comprehensive miRNA profile using high-throughput sequencing technology in peripheral blood lymphocytes from MGN patients compared to healthy controls and reported that 20 kinds of miRNAs levels were increased or decreased in MGN patients compared to the healthy controls [32]. Among these miRNAs, the levels of miR-217 were dramatically downregulated. Based on the report, Li et al. analyzed the expression of plasma and kidney miR-217 in MGN patients and reported the decreased miR-217 expressions in plasma and kidney tissues in MGN patients compared to control patients [33], and also demonstrated that the downregulation of miR-217 may cause podocyte apoptosis via targeting TNF superfamily member 11 (TNFSF11). miR-186 expression was also reported to be downregulated in MGN kidney tissues and to be associated with podocyte apoptosis [34]. On the other hand, Hejazian et al. reported the increase in miR-30c levels from peripheral blood mononuclear cells and plasma miR-186 levels in MGN patients compared to FSGS patients and healthy controls [35]. Although samples analyzed in these two studies were different, further analysis might be required to make conclusion. Rahbar et al. analyzed the expression pattern of the glucocorticoid receptors α and β, and their epigenetic regulators, miR-24, miR-30a and miR-370 in peripheral blood mononuclear cells, and reported that the increase in miR-30a and miR-370 levels as well as the decrease in miR-24 levels were observed in MGN patients compared to FSGS patients, while no significant differences in glucocorticoid receptors α and β expressions were observed between MGN, FSGS and healthy controls, indicating that dysregulation levels of glucocorticoid receptors α and β were not attributed to steroid resistance [36]. Liu et al. found that miR-130-5p potentially regulates PLA2R1 and, thus, investigated the correlation between miR-130-5p and MGN, and reported the decreased expression of miR-130-5p in kidneys of MGN patients [37]. They conducted animal experiments and demonstrated that the decrease in miR-130a-5p expression may increase PLA2R expression, thereby inducing podocyte apoptosis. Zhang et al. revealed the increased expression of urine miR-193a in MGN patients compared to healthy controls, and higher urine miR-193a levels were associated with poor renal survival [38]. Zhou et al. conducted the microarray analysis and reported that serum and urine miR-195-5p, miR-192-3p, miR-328-5p levels and their target genes, PPM1A, RAB1A and BRSK1 may be the potential biomarkers for MGN [39]. Barbagallo et al. analyzed miRNA profile and their target mRNAs from MGN kidney tissues and indicated that let-7a-5p, let-7b-5p, let-7c-5p, let-7d-5p, miR-107, miR-423-5p, miR-516-3p, miR-532-3p and miR-1275 levels were upregulated, and miR-129-3p levels were downregulated in MGN kidney samples compared to unaffected kidney tissues undergoing nephrectomy for renal cancer [40]. They also indicated that these miRNAs may target interleukin 6 and MYC mRNAs, that might be involved in the pathogenesis of MGN, including cell cycle, proliferation and apoptosis.

In spite of the accumulating evidence, most of these reports only analyzed specific miRNAs and there were only three reports in which comprehensive miRNA analysis by microarray was conducted [32,39,40]. In addition, most of these studies analyzing miRNAs have been compared MGN patients to healthy controls, not to other NS diseases, such as MCNS and FSGS, thus these miRNA profile changes might not be directly associated with MGN pathogenesis, but might be the common trend in NS patients. Further validation studies between MGN and other causes of NS is still required to make conclusions.

## 4. Biomarkers in Minimal Change Nephrotic Syndrome and Focal Segmental Glomerulosclerosis

MCNS is the most common disease to cause NS in children. Since renal biopsy is unlikely to be conducted in child MCNS patients without atypical features, such as hematuria and steroid-resistance, non-invasive tools to differentiate MCNS from other causes of NS—especially FSGS, which is another important cause of NS in children—are desired. Recently, Inoue-Torii et al. reported that the levels of urine semaphorin3a are associated with disease activity in MCNS patients [41]. Semaphorin3a is the secreted protein that is involved in several biological functions. In mammalian kidneys, semaphorin3a is expressed in the glomerular podocytes and tubular cells, and is involved in the pathogenesis in podocyte injury. For example, semaphorin3a has been reported to cause podocyte apoptosis through the activation of c-Jun N-terminal kinase (JNK) signaling [42], indicating that semaphorin3a may be identified not only as the biomarker for MCNS patients but also as the therapeutic target in NS. In addition to semaphorin3a, several possible biomarkers for the diagnosis as well as the pathogenesis of MCNS have been reported. CD80 (also known as B7.1) is one of the possible markers. CD80 is the dendrite-associated receptor which induces co-stimulatory signaling in T cells [43] and is a transmembrane glycoprotein expressed in podocytes in the kidneys [44]. Urine CD80 levels were reported to be upregulated in the relapse of MCNS [45]. The administration of lipopolysaccharide (LPS) into mice caused rapid onset of proteinuria with CD80 expression in podocytes, and the onset of proteinuria caused by LPS was prevented when injected into CD80 knockout mice [46]. In addition, serum from MCNS patients under relapse stimulated CD80 expression in cultured podocyte, suggesting that CD80 might be involved in the pathogenesis in MCNS [44]. Interleukin 13 (IL-13), that is the cytokine secreted from Th2 cells, is another potent biomarker in MCNS. The increase in IL-13 mRNA in CD4(+)-CD8(+) T cells from child steroid-sensitive NS patients was reported [47]. Subsequently, serum IL-13 levels were found to be increased in child NS patients [48]. Importantly, there are several reports indicating that IL-13 might cause podocyte injury and MCNS. For example, it is reported that IL-13 decreased the function of glomerular filtration barrier in the dose dependent manner [49]. In addition, overexpression of IL-13 caused MCNS-like glomerulopathy in rats, suggesting the possible involvement in the pathogenesis for MCNS [50]. Hemopexin was another possible marker for the diagnosis of MCNS. The decrease in serum hemopexin with higher protease activity in MCNS under relapse was reported [51]. In addition, the injection of hemopexin into rats caused reversible proteinuria [51]. Since hemopexin has been reported to induce nephrin-dependent actin cytoskeleton reorganization in podocytes [52], hemopexin might be another factor to be involved in the pathogenesis of MCNS. Finally, angiopoietin-like 4 (Angptl-4), a secreted glycoprotein, has been reported as another potent marker [53]. Angptl-4 is highly expressed in the adipose tissue and liver, and is known to be associated with tumor metastasis [54]. It is reported that the expression of angptl-4 in serum and podocytes in MCNS as well as the rodent podocytes under the treatment with LPS or puromycin aminonucleoside (PAN) were increased [53]. In addition, podocyte-specific overexpression of angptl-4 in rats caused nephrotic range of proteinuria. Interestingly, adipose tissue-specific angptl-4 overexpression did not cause proteinuria while there were increased levels of circulating angptl-4, indicating the importance of podocyte angptl-4 expression under NS [53]. However, subsequent reports from same group revealed that the increased levels of angptl-4 were also observed in other NS patients, including FSGS and MGN [55]. Although these several biomarkers have been reported, further analysis is still required to make conclusions.

FSGS is another important disease to cause NS. It is known that podocyte damage plays the essential role in the development of FSGS. There are varieties of different causes of FSGS, primary and secondary FSGS, including genetic, adaptive, *APOL1*-associated, virus-associated, and medication/toxin-associated FSGS [56]. However, the underlying causes are still unknown in most cases, which are defined as primary or idiopathic FSGS. As described previously, the circulating factors, which may cause podocyte injury or increase the glomerular permeability, are believed to be the pathogenetic for FSGS. Wei et al. reported suPAR as the circulating factor to cause FSGS. The urokinase-type plasminogen activator receptor (uPAR) has been known to have important roles for cell migration. uPAR might be shed from cell membrane, called suPAR, which might cause podocytopathy as circulating factors. Indeed, it is reported that the overexpression of uPAR in podocytes as well as the administration of suPAR in mice caused proteinuria [57]. Increased suPAR levels in FSGS patients were also reported in meta-analysis reports [58,59]. However, several conflicting results have also been reported [60,61,62]; thus, it is still controversial whether suPAR is involved in the pathogenesis of FSGS. Therefore, further exploration of the pathogenesis as well as the specific biomarkers are still required. In the point of miRNAs, there is accumulating evidence as the biomarker in MCNS as well as FSGS (Table 3).

## 5. miRNAs in Minimal Change Nephrotic Syndrome and Focal Segmental Glomerulosclerosis

Lu et al. analyzed renal tissues from child NS patients in different subtypes, and indicated that the expressions of miR-150 were lower in MCNS compared to some other types of NS, suggesting the possibility that miR-150 might be the indicator to differentiate MCNS from other types of NS [22]. On the other hand, Qi et al. reported increased miR-150 expressions in kidneys from FSGS patients compared to healthy controls [67]. They also indicated the protective effect of miR-150 inhibitor for doxorubicin-induced FSGS mouse model through anti-fibrosis and anti-inflammation mechanisms. These reports might reflect the protective effect of miR-150 inhibition against podocyte injury.

Yang et al. analyzed renal tissues from FSGS patients and indicated increased expression of miR-135a [64]. They also indicated the increased expression of miR-135a in doxorubicin-induced mouse kidneys. In addition, they demonstrated that the ectopic expression of miR-135a resulted in severe podocyte injury and disarray of podocyte cytoskeleton while podocyte-specific expression of transient receptor potential canonical 1 (TRPC1) was able to reverse the effect of miR-135a in cultured podocytes, demonstrating the dependency of TRPC1. In another study, Zhang et al. compared plasma miRNAs from FSGS patients, MGN patients, diabetic nephropathy (DN) patients and healthy controls, and revealed that plasma miR-125b, miR-186 and miR-193a-3p levels were higher in FSGS patients than in patients with other diseases [65]. In addition, plasma miR-125b and miR-186 levels declined markedly in patients with FSGS with complete remission while there was no decrease in FSGS patients with steroid resistant. Importantly, plasma miR-125b and miR-186 levels were not upregulated in MGN and DN patients, suggesting the possibility of these miRNAs as the specific biomarkers for FSGS. Nevertheless, there was no comparison between MCNS and FSGS, which may be the limitation in the study. Interestingly, plasma miR-186 levels, but not miR-125b levels, were positively correlated with degrees of proteinuria in FSGS patients [65]. Meanwhile, as described previously, Hejazian et al. reported the increase in plasma miR-186 levels in MGN patients, not in FSGS patients, compared to healthy controls, indicating that it is still controversial [35].

Xiao et al. reported, using qPCR-based high-throughput miRNA profile, that plasma miR-17, miR-451, miR-106a and miR-19b levels were downregulated in FSGS patients compared to healthy controls. They also indicated that plasma miR-17, miR-451 and miR-106a levels were associated with remissions of FSGS [68]. They suggested that miR-106a might target phosphatase and tensin homolog (PTEN), Bcl-2-like protein 11 (BCL2L11) and C-X-C motif chemokine ligand 14 (CXCL14), thereby regulating podocyte apoptosis. Although they compared FSGS patients to other diseases, including IgA nephropathy, MGN and mesangial proliferative glomerulonephritis, MCNS patients were not included in the study.

For the differential diagnosis of MCNS and FSGS, miRNA comparison between these diseases is required. Gebeshuber et al. reported that miR-193a expressions in FSGS kidneys were upregulated compared to the kidneys from healthy controls and other glomerular diseases, including MCNS [70]. They reported that the overexpression of miR-193a in mice rapidly induced FSGS. miR-193a may target Wilms’ tumor protein (WT1) necessary for podocyte homeostasis. They concluded that miR-193 might be involved in the pathogenesis of FSGS and be the therapeutic target. Cai et al. reported that serum miR-192 and miR-205 levels were higher in FSGS patients compared to MCNS patients [71]. Levels of serum miR-192 and miR-205 were correlated with proteinuria in FSGS patients while miR-192 levels, not miR-205 levels, were correlated with proteinuria in MCNS. In addition, serum miR-192 levels were correlated with interstitial fibrosis in FSGS patients. In a comprehensive miRNA analysis between MCNS and FSGS, Ramenzani et al. compared plasma and urine miRNAs from FSGS patients, MCNS patients and healthy controls using Affymetrix GeneChip miRNA 3.0 arrays, and reported that plasma miR-30b, miR-30c, miR-34b, miR-34c and miR-342 levels, as well as urine miR-1225-5p levels, were higher in MCNS patients compared to FSGS patients and controls [63]. On the other hand, urine miR-1915 and miR-663 levels were lower and urine miR-155 levels were higher in FSGS patients compared to MCNS patients and the controls. Taken together, these results suggest that plasma miR-30, miR-34 and miR-342 as well as urine miR-1225 might be the potent diagnostic biomarkers for MCNS while urine miR-1915, miR-663 and miR-155 might be the potent biomarkers for FSGS.

Among these markers, miR-30 is worth focusing on since there are a lot of publications under the analysis of NS. As described, several reports indicated the increase in serum or urine miR-30 levels [18,20,25] in NS patients as well as the higher serum miR-30 levels in MCNS compared to FSGS [63]. How about the miR-30 expression in FSGS? Hajazian et al. reported no significant difference in plasma miR-30c levels between FSGS patients and healthy controls [35]. On the other hand, Wu et al. reported that miR-30 family, miR-30a, miR-30b, miR-30c, miR-30d and miR-30e, in FSGS glomeruli were all downregulated compared to healthy controls [66]. Zhang et al. analyzed urine miRNAs in FSGS patients by the TaqMan Low Density Array as well as RT-qPCR and indicated that urine miR-196a, miR-30a-5p and miR-490 levels were higher in FSGS patients compared to the remission period [69], indicating that these miRNAs are associated with disease activity of FSGS. In addition, urine miR-30a-5p levels were shown to predict the response to steroid therapy in FSGS patients, indicating the possible biomarker for disease monitoring. Wang et al. reported that urine miR-10a and miR-30d levels in FSGS patients were higher compared to healthy controls [72]. Taken together with these reports, urine miR-30 levels, not serum miR-30 levels, may be increased in FSGS patients while serum miR-30 levels may be increased in MCNS patients. Increased urine miR-30 levels in FSGS patients might be the reflection of the increase in urine podocyte detached from the glomeruli. In this case, we may explain the expression pattern difference between reports, diseases and types of analyte. miR-30 might be indispensable for the maintenance of podocyte integrity since miR-30 family has been reported to be podocyte protective [73]. For example, Wu et al. reported that miR-30 family may protect from apoptosis by targeting Notch1 and p53 [66]. miR-30 family may also promote actin fiber stability in podocytes through the regulation of calcium/calcineurin signaling by targeting transient receptor potential cation channel subfamily C member 6 (TRPC6), protein phosphatase 3 catalytic subunit alpha (PPP3CA), protein phosphatase 3 catalytic subunit beta (PPP3CB), protein phosphatase 3 regulatory subunit alpha (PPP3R1) and nuclear factor of activated T cells 3 (NFATC3) [74,75]. Lang et al. also demonstrated, using podocyte-specific miR-30 knockdown mice, that miR-30 may prevent uPAR-integrin β3 (ITGB3) signaling through calcineurin-NFATC pathway, thereby protect podocytes [76]. miR-30 has also been reported to be podocyte protective against aldosterone-induced podocyte apoptosis and mitochondrial dysfunction through targeting BCL2 interacting protein 3 like (BNIP3L) [77]. Based on these reports, targeting miR-30 might be the therapeutic target for podocyte protection as well as diagnostic biomarkers for NS.

## 6. Future Requirement for Further Exploration

In spite of the accumulating evidence, there are still several limitations. First, there are multiple pathological conditions even in the same disease. For example, under the analysis of FSGS, there are varieties of different types of FSGS, primary and secondary FSGS, including genetic, adaptive, *APOL1*-associated, virus-associated, medication/toxin-associated FSGS [56], thus FSGS contains different types of etiology, that make the systematic analysis more difficult. There are also different types of variant in the point of pathology among FSGS patients, including collapsing, tip, perihilar, cellular and not otherwise specified (NOS) [56]. Since these different causes of FSGS and different pathological variant might have the different serum and urine miRNA profiles, we need to keep in mind that FSGS contains these varieties of disease area and, thus, it is sometimes hard to interpret the data.

Second, there are two miRNA arms from the same precursor. miRNAs from 5′ and 3′ arms were annotated using the -5p and -3p suffixes [78]. Normally, one miRNA arm is served as the dominant miRNA while another miRNA arm is usually degraded [79]. However, in some cases, both arms are present and target different mRNAs [80,81]. It is also reported that -5p and -3p miRNAs might be biologically different in terms of stability and functionality [82]. In addition, the preference of -5p and -3p is not always consistent depending on tissue types, stages of development and different diseases, suggesting the presence of some regulatory mechanisms [83]. Therefore, miR-5p/miR-3p ratio validations in NS might be another possible biomarkers. Considering these published reports, some reports distinguished -5p and -3p, while others did not. It would be better to account the -5p and -3p as different biomarkers, and to analyze, respectively.

Finally, there are a lot of different results that have come from the various studies as described. There might be several reasons. One of the most essential reasons for this point might be that not so many reports conducted the comprehensive miRNA analysis for example by microarray analysis, and most of the published reports focused on specific miRNAs known to be associated with NS. In addition, different analyte, including serum, plasma, peripheral blood mononuclear cells, urine and kidney tissues, as well as the different comparison, such as healthy controls or other NS diseases might cause the different profile results. The analysis in child NS or adult NS patients may cause the different results as well. In addition, sample size were very low in some reports. It would be required to understand these limitations and it would be better to conduct more comprehensive and large-scale miRNA analysis in NS patients.

## 7. Conclusions

As reviewed, there is accumulating evidence that indicates potential tools of miRNAs as biomarkers for NS. Nevertheless, further analysis with higher numbers and appropriate measurements of miRNAs are still required to assess the reliable tool of miRNAs in the diagnosis, disease activity and prognosis. They are promising since these miRNA data might be used not only for the diagnostic or prognostic biomarkers but also for understanding the pathogenesis of podocyte injury and the specific etiologies of MCNS, FSGS and MGN.

## Figures and Tables

**Table 1:** MiRNAs in nephrotic syndrome.

miRNA	Analyte	Disease	Comparison	Levels	Feature	Reference
miR-30a-5p miR-151-3p miR-150 miR-191 miR-19b	serum	Child NS	healthy control	↑		[18]
miR-30a-5p	urine	Child NS	healthy control	↑		[18]
miR-17-5p	peripheral blood	Child NS	healthy control	↑		[19]
miR-194-5p miR-146b-5p miR-378a-3p miR-23b-3p miR-30a-5p	urine	Child NS	healthy control	↑	All decrease in clinical remission period miR194-5p and miR-23b-3p positively correlate with urine protein	[20]
miR-503	serum	Child NS	healthy control	↓		[21]
miR-191	kidney	Child NS	healthy control	↑		[22]
miR-151-3p	kidney	Child NS	healthy control	↓		[22]
miR-638	urine	NS	non-NS control	↓		[23]
miR-16-1	serum	NS	healthy control	↓		[24]
miR-181a miR-210 miR-30a miR-942 miR-192 miR-586	serum	NS	healthy control	↑	miR-30a: higher in steroid resistant patients	[25]
miR-181a	serum	NS	healthy control	↑		[26]

NS: nephrotic syndrome; **↑**: increase; **↓**: decrease.

**Table 2:** MiRNAs in membranous glomerulonephropathy.

miRNA	Analyte	Comparison	Levels	Mechanism	Reference
miR-217	Peripheral blood lymphocytes	healthy control	↓		[32]
miR-217	plasma kidney	healthy control	↓	podocyte apoptosis	[33]
miR-30c	Peripheral blood mononuclear cells	FSGS healthy control	↑		[35]
miR-186	plasma	FSGS healthy control	↑		[35]
miR-30a	Peripheral blood mononuclear cells	FSGS	↑		[36]
miR-24	Peripheral blood mononuclear cells	FSGS healthy control	↓		[36]
miR-370	Peripheral blood mononuclear cells	FSGS healthy control	↑		[36]
miR-186	kidney	healthy control	↓	podocyte apoptosis	[34]
miR-130-5p	kidney	control patients	↓	PLA2R- podocyte apoptosis	[37]
miR-193a	urine	healthy control	↑		[38]
miR-195-5p miR-192-3p	Peripheral blood mononuclear cells urine	healthy control	↑		[39]
miR-328-5p	Peripheral blood mononuclear cells urine	healthy control	↓		[39]
let-7a-5p let-7b-5p let-7c-5p let-7d-5p miR-107 miR-423-5p miR-516-3p miR-532-3p miR-1275	kidney	normal kidney from renal cell carcinoma	↑		[40]
miR-129-3p	kidney	normal kidney from renal cell carcinoma	↓		[40]

FSGS: focal segmental glomerulosclerosis; PLA2R: phospholipase A2 receptor; **↑**: increase; **↓**: decrease.

**Table 3:** MiRNAs in minimal change nephrotic syndrome and focal segmental glomerulosclerosis.

miRNA	Analyte	Disease	Comparison	Levels	Mechanism	Reference
miR-150	kidney	Child MCNS	other NS types	↓		[22]
miR-30b miR-30c miR-34b miR-34c miR-342	plasma	MCNS	FSGS healthy control	↑		[63]
miR-1225-5p	urine	MCNS	FSGS healthy control	↑		[63]
miR-135a	kidney	FSGS	normal control	↑	TRP channel 1-disarray of podocyte cytoskeleton	[64]
miR-125b miR-186 miR-193a-3p	plasma	FSGS	MGN DN healthy control	↑		[65]
miR-155	urine	FSGS	MCNS healthy control	↑		[63]
miR-1915 miR-663	urine	FSGS	MCNS healthy control	↓		[63]
miR-30a miR-30b miR-30c miR-30d miR-30e	kidney	FSGS	healthy control	↓		[66]
miR-150	kidney	FSGS	healthy control	↑	fibrosis inflammation	[67]
miR-17 miR-451 miR-106a miR-19b	plasma	FSGS	healthy control	↓	podocyte apoptosis	[68]
miR-196a miR-30a-5p miR-490	urine	FSGS	remission period	↑		[69]
miR-193a	kidney	FSGS	healthy controls other glomerular diseases	↑	regulation of WT1	[70]
miR192 miR-205	serum	FSGS	MCNS	↑		[71]
miR-10a miR-30d	urine	FSGS	healthy control	↑		[72]

MCNS: minimal change nephrotic syndrome; FSGS: focal segmental glomerulosclerosis; NS: nephrotic syndrome; DN: diabetic nephropathy; WT1: Wilm’s tumor-1; **↑**: increase; **↓**: decrease.

## Data Availability

Data sharing not applicable.

## Abbreviations

NS	nephrotic syndrome
MCNS	minimal change nephrotic syndrome
FSGS	focal segmental glomerulosclerosis
MGN	membranous glomerulonephropathy
suPAR	soluble form of the urokinase receptor
miRNA	microRNA
3′-UTR	three prime untranslated region
PLA2R1	phospholipase A2 receptor 1
THSD7A	thrombospondin type-1 domain-containing 7A
NELL-1	neural epidermal growth factor-like 1 protein
TNFSF11	TNF superfamily member 11
JNK	c-Jun N-terminal kinase
LPS	lipopolysaccharide
IL-13	interleukin 13
Angptl-4	angiopoietin-like 4
uPAR	urokinase plasminogen activator receptor
PAN	puromycin aminonucleoside
TRPC1	transient receptor potential canonical 1
DN	diabetes nephropathy
PTEN	phosphatase and tensin homolog
BCL2L11	Bcl-2-like protein 11
CXCL14	C-X-C motif chemokine ligand 14
WT1	Wilms’ tumor protein
TRPC6	transient receptor potential cation channel subfamily C member 6
PPP3CA	protein phosphatase 3 catalytic subunit alpha
PPP3CB	protein phosphatase 3 catalytic subunit beta
PPP3R1	protein phosphatase 3 regulatory subunit alpha
NFATC3	nuclear factor of activated T cells 3
ITGB3	integrin β3
BNIP3L	BCL2 interacting protein 3 like
NOS	not otherwise specified
